# Adipocyte Phenotype Flexibility and Lipid Dysregulation

**DOI:** 10.3390/cells11050882

**Published:** 2022-03-03

**Authors:** Kyle J. Preston, Rosario G. Scalia, Michael V. Autieri

**Affiliations:** The Lemole Center for Integrated Lymphatic Research, Lewis Katz School of Medicine, Temple University, Philadelphia, PA 19140, USA; kyle.preston@temple.edu (K.J.P.); rosario.scalia@temple.edu (R.G.S.)

**Keywords:** adipose tissue, phenotype, hypertrophy, lipolysis, lipid buffering, hypoxia, inflammation, postprandial

## Abstract

The prevalence of obesity and associated cardiometabolic diseases continues to rise, despite efforts to improve global health. The adipose tissue is now regarded as an endocrine organ since its multitude of secretions, lipids chief among them, regulate systemic functions. The loss of normal adipose tissue phenotypic flexibility, especially related to lipid homeostasis, appears to trigger cardiometabolic pathogenesis. The goal of this manuscript is to review lipid balance maintenance by the lean adipose tissue’s propensity for phenotype switching, obese adipose tissue’s narrower range of phenotype flexibility, and what initial factors account for the waning lipid regulatory capacity. Metabolic, hypoxic, and inflammatory factors contribute to the adipose tissue phenotype being made rigid. A better grasp of normal adipose tissue function provides the necessary context for recognizing the extent of obese adipose tissue dysfunction and gaining insight into how pathogenesis evolves.

## 1. Introduction

One in four deaths in the western world can be attributed to cardiovascular disease [1]. The majority of cardiovascular diseases are vascular in origin, and, despite advances in therapeutics and lifestyle adjustments, vascular disease prevalence is increasing throughout the world. This trend is expected to worsen as our population ages and younger individuals adopt a more sedentary lifestyle [2]. Metabolic syndrome is a clustering of medical conditions that include increased abdominal adiposity and hyperlipidemia, which predispose patients to systemic inflammation and atherosclerotic vascular disease [3]. While vascular and metabolic diseases, such as insulin resistance and diabetes, are diagnosed independently, it is clear that they share risk factors and promote the pathogenesis of the other [4,5,6,7,8]. As a result of the complex interconnectedness of cardiometabolic diseases, patients with multiple pathologies are especially difficult to treat. To this end, a multifactorial approach is required to better understand the molecular and cellular mechanisms of cardiometabolic diseases to combat this global pandemic. The treatment of patients with substantiated cardiometabolic diseases (increasing case clearance) and the prevention of new cases developing (decreasing case appearance) are major socieoeconomic as well as major medical concerns. With this shared etiology and process complexity, a better understanding of relevant cellular phenotypes is essential to make these goals more attainable.

White adipose tissue is a secretory organ, and a unique driver of metabolism and cellular phenotypes throughout the body due to its influence over blood lipid concentration via lipid storage and secretion. Alterations in lipid exposure cause phenotypic shifts in insulin-sensitive cell types, such as hepatocytes, pancreatic beta cells [9], cardiomyocytes [10], and skeletal muscle cells [11,12]. The same is true of anatomically ubiquitous cells not typically described as metabolic in nature. For example, the phenotypic fate of immune cells [13,14], endothelial cells [15], and smooth muscle cells [16] is determined in part by energetic substrates such as plasma free fatty acids. In this way, a wide variety of cell types throughout the body take their phenotypic cues from the adipose tissue phenotype and, in particular, the balance between lipolysis and lipid clearance. Therefore, the preservation of white adipocyte phenotypic flexibility is central for maintaining cardiometabolic health. Consequently, adipocyte dysregulation is an under-characterized and overlooked point of therapeutic intervention with untapped potential.

In addition to stromal cells, healthy white adipose tissue comprises adipocytes that regularly switch between prioritizing lipid mobilization and storage depending on the energy demands of tissues and the concentration of energetic substrates in the blood. Therefore, feeding cycles are a large determinant of adipocyte behavior. Recent studies of the postprandial phase emphasize the dynamism of adipose phenotype switching and suggest adipose tissue dysregulation is an early event in cardiometabolic pathogenesis. This evidence suggests recurrent phases of adipocyte dysfunction raise the blood lipid concentration, which then acts as a pathogenic positive feedforward mechanism, ultimately resulting in hyperlipidemia and a predisposition for cardiometabolic diseases. This discussion will characterize how phenotypically flexible versus rigid white adipose tissue respond to fasting/feeding cycles and will identify what early cellular events predispose adipocytes to pathological behavior. The authors recognize that adipose tissue phenotypes may be influenced by epigenetic and transgenerational factors [17,18] and also that the browning of adipose tissue is a relevant form of adipocyte phenotype shifting. An entire review article could be devoted to that important topic alone. However, we will limit the focus of this review to the pathological loss of flexibility that occurs in the white adipocytes of an individual organism. A goal of this report is the highlighting of pathways and potential targets for ameliorating lipid homeostasis. A better recognition of eroding adipose tissue phenotype flexibility will improve treatment strategies for patients with dysfunctional adipose tissue and will inform techniques toward preventing pathology development.

For our search methodology, we searched Pubmed for the terms “lipolysis”, “adipocyte lipid storage”, “postprandial adipose”, and “hypertrophic adipocytes” using relevance to adipocyte physiology as the only exclusion criteria. We did not limit publication age, so seminal papers in the field could be included.

## 2. Flexible Adipose Tissue

### 2.1. Lipid Secretion

**Figure 1:**
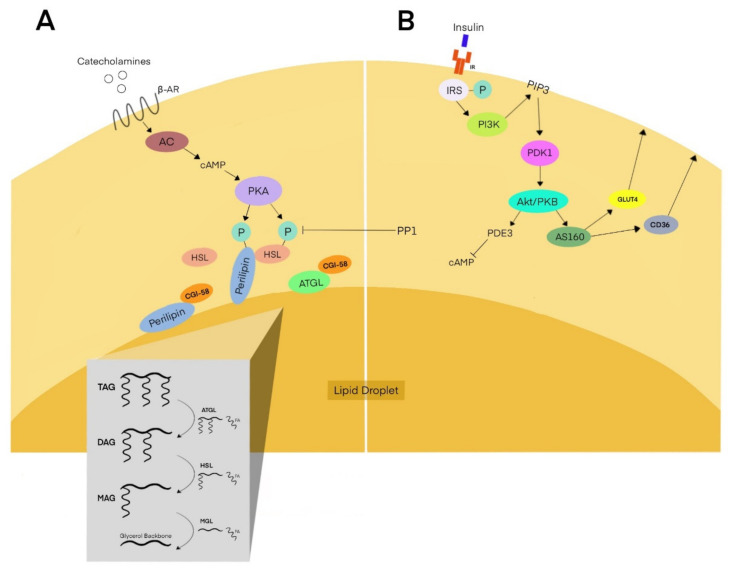
Lipid secretion and storage model. (**A**): Catecholamine stimulation of lipolysis results in cAMP accumulation, PKA activation, and phosphorylation of lipolytic enzymes. A Insert: Stepwise hydrolysis reactions mediated sequentially by ATGL, HSL, and MGL. (**B**): Insulin stimulates the PI3K/Akt pathway to decrease lipolysis via lowered cAMP and activation of protein phosphatase 1. Simultaneously, AS160 downstream of insulin promotes CD36 and GLUT4 translocation from the cytosol to the cell membrane.

The fasted condition is characterized by an imbalance in tissue energy demands and energetic substrates supplied to tissues by the blood. To compensate for this imbalance, white adipocytes undergo lipolysis, wherein triacylglycerides (TAG) stored in lipid droplets are sequentially hydrolyzed in a series of enzymatic reactions mediated by adipose triglyceride lipase (ATGL), hormone-sensitive lipase (HSL), and monoacylglycerol lipase (MGL) to produce and secrete non-esterified free fatty acids into the blood for delivery to tissues. The regulation of lipolysis occurs at the transcriptional, post-translational, and neuroendocrine levels in processes that have recently been reviewed [19,20,21,22]. The lipolytic process is summarized in the following section and Figure 1A.

The catecholamine ligation of beta-adrenergic G-protein coupled receptors on the adipocyte plasma membrane is the primary stimulus for lipolysis, although natriuretic peptides also trigger lipolysis. Following catecholamine stimulation, activated adenylyl cyclase (AC) generates an accumulation of the second messenger cyclic AMP (cAMP). cAMP is a key regulator of stimulated adipocyte increments in lipolytic kinetics, as it binds to and activates protein kinase A (PKA) [23,24,25]. PKA exerts its pro-lipolytic activity by direct and indirect mechanisms. PKA activates HSL by phosphorylating it on three serine residues (S552, S649, and S650) [26]. In addition to HSL, PKA also phosphorylates perilipin1, a lipid-droplet-associated scaffold protein that, under basal conditions, shields lipid droplets from hydrolytic lipases [26,27] and binds the ATGL peptide co-regulator comparative gene identification-58 (CGI-58), also known as alpha/beta-hydrolase domain-containing protein 5 (ABHD5). PKA targets and phosphorylates six perilipin1 serine residues (S81, S222, S276, S433, S492, and S517) [26]. After PKA phosphorylation, perilipin1 undergoes conformational changes that significantly increase the efficiency of lipolysis: (1) perilipin1 dissociates from the lipid droplet, exposing its TAG to hydrolytic lipases [28,29]; (2) CGI-58 disengages from perilipin1 and then binds and activates ATGL [30,31]; (3) perilipin1 provides the necessary scaffolding to facilitate the colocalization of lipases and their associated proteins to the lipid droplet [32,33,34].

ATGL hydrolyzes TAG to form diacylglycerol (DAG) and fatty acid [35]. TAG hydrolysis by ATGL is regarded as the rate limiting step of lipolysis due to its relatively slow reaction rate compared to the HSL-mediated hydrolysis of DAG to monoacylglycerol (MAG) [36]. The PKA phosphorylation of HSL activates HSL hydrolytic activity. While HSL has been shown to hydrolyze TAG, DAG, cholesteryl esters, and retinyl esters, its primary substrate is DAG [37]. This preference for DAG hydrolysis is corroborated by experiments performed in HSL-null mice, which demonstrate an accumulation of DAG, not TAG, in mouse adipocytes [38]. The hydrolysis of DAG by HSL releases a fatty acid and forms MAG [37,39,40], which is hydrolyzed by MGL to form fatty acids and glycerol. This sequence is summarized in the Figure 1A insert. The authors acknowledge that alternative lipases contribute to lipolysis (reviewed elsewhere—[19,20,21,22]) but have chosen to focus on the canonical ATGL and HSL, as they have been shown to be responsible for nearly 90% of TAG hydrolysis [41].

More work is needed to gain a deeper understanding into the mechanisms governing the export of non-esterified fatty acids (NEFAs). The predominant theory is that fatty acids liberated by lipases require molecular chaperones for transport between the lipid droplet and the plasma membrane, a role filled by fatty acid binding proteins (FABPs) [42]. In adipocytes, FABP4, also known as adipocyte protein 2 (aP2) is known to interact with both HSL [43] and CGI-58 [44] at the lipid droplet to increase lipolytic activity. The specific mechanism by which FABP4 transports fatty acids from the lipid droplet to the plasma membrane remains to be fully elucidated. The complexity of the adipocyte lipolytic process assures many points of control and opportunities for therapeutic intervention.

In summation, the catecholamine stimulation of adipocytes initiates signaling events that promote the translocation of lipolytic enzymes to the lipid droplet and lipid-droplet-associated protein conformational changes, which facilitate lipase–lipid interactions. TAG, DAG, and MAG are hydrolyzed by ATGL, HSL, and MGL, respectively, wherein the first two reactions produce a free fatty acid and the third and final reaction produces fatty acid and glycerol. How liberated free fatty acids are exported remains to be fully elucidated; however, it is expected that this process relies on chaperones such as FABP4.

### 2.2. Lipid Storage

In the postprandial state, adipocytes demonstrate a phenotypic dynamism by shifting away from lipolysis and towards lipid storage. Lipolysis is downregulated by insulin signaling, which mediates cAMP-dependent and independent mechanisms. The canonical lipid storage signaling pathway includes insulin binding its receptor, increasing the insulin receptor tyrosine kinase activity, which, in turn, phosphorylates insulin receptor substrate (IRS) proteins [45]. IRS activates phosphatidylinositol 3-kinase (PI3K), which generates phosphatidylinositol 3,4,5-triphosphate (PIP3) in the cell membrane. Accumulated PIP3 stimulates phosphoinositide-dependent kinase-1 (PDK1) [46], which then activates Akt/PKB. Akt is thought to then phosphorylate phosphodiesterase 3 (PDE3), which degrades cAMP, reducing PKA activity and interrupting the phosphorylation of perilipin1 and HSL (Figure 1B). Consequently, ATGL and HSL hydrolytic activity are attenuated [47]. In addition to this mechanism, insulin activates protein phosphatase 1, which directly dephosphorylates HSL [48] and may also downregulate ATGL mRNA [49], which could partially explain the observation that ATGL is suppressed during refeeding [35].

In addition to blocking lipolytic activity, insulin also stimulates nutrient import and lipid storage [50]. Downstream of the PI3K/Akt/PKB pathway, Akt phosphorylates the Akt substrate of 160 kDa (AS160) to facilitate the translocation of glucose transporter-4 (GLUT4) from the cytosol to the plasma membrane to import circulating glucose from the blood (Figure 1B) (reviewed in [51]). Intracellular glucose is recognized to undergo multiple metabolic fates. A small portion of glucose is converted to glycogen for storage [52]. Glycolysis also generates glycerol 3-phosphate (G3P), which may be used as the backbone for triacylglycerol. Glucose may also be metabolized to acetyl-coA for de novo lipid synthesis (reviewed in [53,54]). Finally, depending on need, glucose may also be converted to lactate, which may act in an anti-lipolytic, autocrine fashion or be exported for use by other cell types (reviewed in [55,56,57]).

In the postprandial phase, dietary fat is largely transported in the blood by lipoproteins, such as very-low-density lipoprotein and chylomicrons. White adipose tissue has the unique ability of removing lipids from the blood for storage in a process that has historically been described as a lipid-buffering capacity [58]. Insulin stimulates lipoprotein lipase (LPL) activity, enabling the hydrolysis of lipoprotein-bound TAGs that either enter adipocytes [59] for re-esterification or remain in the blood in a phenomenon called lipid spillover [60]. Fatty acids cross the adipocyte plasma membrane both by passive diffusion [61] and active protein transport [62,63]. Cluster of differentiation-36 (CD36), also known as fatty acid translocase, is a scavenger receptor capable of binding many ligands, and is responsible for a large percentage [61,64] of fatty acid active transport [65]. Increases in the surface expression of CD36 are rapid due to roughly 50% of CD36 being stored in the cytosol until recruitment to the membrane for lipid internalization (reviewed in [66]); however, hyperlipidemia in *ob*/*ob* mice has been shown to increase CD36 mRNA [67]. Like GLUT4, CD36 is downstream of the insulin/AS160 signaling pathway (Figure 1B). Fatty acid transport proteins facilitate this process, but the exact mechanism remains unclear. Once in the cell, fatty acids are esterified in a series of reactions mediated by acyl transferases, including lipids and diacylglycerol acyltransferases (DGAT) [68]. Resultant TAGs are stored in adipocyte lipid droplets. When taken together, flexible adipose depots are nimble metabolic regulators and secretory organs. Adipocyte flexibility is exquisitely regulated and an attribute of healthy adipose tissue. Conversely, unhealthy adipose tissue is characterized by rigid adipocytes, which has multiple negative ramifications for the organism as a whole.

In response to feeding, insulin simultaneously applies a break on adipocyte lipolysis and accelerates adipocyte lipid clearance from the blood. Insulin’s anti-lipolytic actions includes decreasing cAMP/PKA activity and increasing protein phosphatase-1 activity. The lipid-storage-promoting effects of insulin include increasing LPL activity and CD36 translocation to the adipocyte membrane. Maintaining the adipose tissue’s lipid-buffering capacity is critical for whole-body lipid homeostasis.

## 3. Rigid Adipose Tissue

### 3.1. Lipid Mobilization Dysfunction

In obesity, enlarged adipose depots comprising a heterogenous mixture of normal-sized and hypertrophic adipocytes lose their ability to maintain lipid homeostasis. The phenotype shifting between fasting and fed states observed in the adipose tissue of lean subjects is lost. Instead, obese adipose tissue responds weakly to catecholamine stimulation and is described as resistant to insulin’s anti-lipolytic effects. With respect to lipid mobilization, the adipose tissue appears inert and phenotypically rigid.

The hyperlipidemia associated with obesity is a risk factor for a wide range of poor cardiac, vascular, and metabolic outcomes and, as such, a large effort has been made to identify the source of the pathologically elevated blood NEFAs and TAGs. Hypertrophic adipocytes, a hallmark of obese adipose tissue, are widely regarded as hyperlipolytic and the presumptive main culprit. Resistance to the anti-lipolytic effects of insulin is the primary explanation for the increased basal lipolytic rate of rigid adipose tissue [69,70]. Insulin’s effects on lipolysis are multi-modal and, therefore, many intermediate points between insulin receptor ligation and decreased lipolysis may be disrupted. A decreased insulin receptor density and diminished insulin receptor tyrosine kinase activity relative to normal-sized counterparts may partly explain adipose tissue insulin resistance [71,72,73,74]. Insulin’s downstream protein targets are also altered in dysfunctional adipocytes. For example, perilipin1, the lipid-droplet-associated protein responsible for shielding lipid droplet TAGs from lipases, has been shown to be decreased relative to the fat cell size in obese adipose tissue [75].

**Figure 2:**
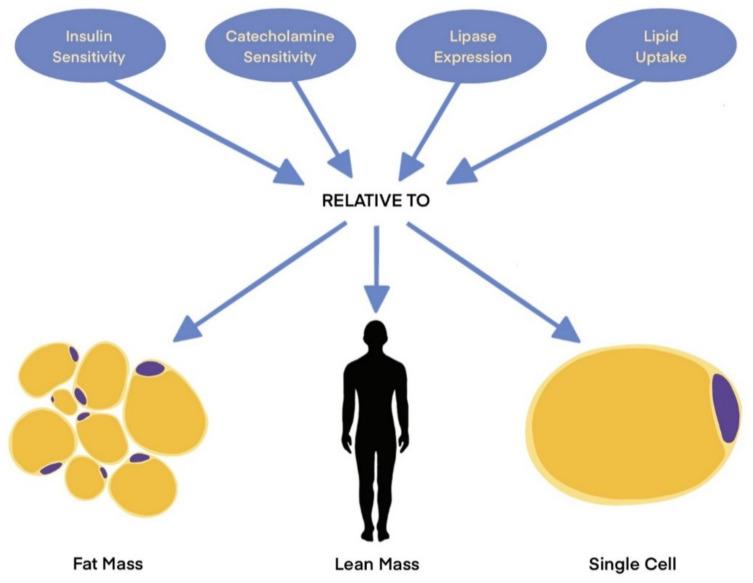
Metabolic variable interpretation is dependent on anatomical context. NEFA secretion, for example, is low relative to fat mass but high relative to lean mass in obese individuals. Imprecise language describing data may lead to confusion.

Comparisons of metabolic variables, such as insulin sensitivity and the lipolytic rate between lean and obese adipose tissue, are obfuscated by differences in total adipose mass [76]. Insulin clamp studies testing insulin sensitivity in lean and obese subjects clearly demonstrate that an equal dose of insulin elicits a stronger response from the lean cohort. Add to this observation the close association of hyperlipidemia, hyperinsulinemia, and obesity, and it is easy to understand the insulin-resistant, hyperlipolytic characterization ascribed to the obese adipose tissue that is so prevalent in the literature. Nevertheless, several studies challenge the notion that this paradigm is always true. Experiments conducted with stable isotope fatty acid tracers [77,78] have shown that the rate of NEFA secretion in obese individuals is high relative to their lean tissue mass, but low relative to their fat mass, when compared to lean individuals following an overnight fast [77]. An increased adiposity coincided with a decreased NEFA release from fat; however, this drop in lipolytic rate was not sufficient to ameliorate lipid metabolism due to the large increase in fat mass. These data are consistent with the observation that both ATGL and HSL are downregulated in obese adipose tissue [79,80]. In addition to lower NEFA mobilization out of the cell, fatty acid uptake by the obese adipose tissue is diminished relative to lean adipose tissue in the postprandial phase [78]. Finally, a recent report [81] has shown that insulin secretion is determined by adiposity before the onset of insulin resistance. These studies provide both revelations about adipose tissue metabolism and highlight how anatomical context alters data interpretation (Figure 2). Taken together, these data suggest that, under certain circumstances, lipid transport in and out of obese adipocytes is depressed whereas insulin sensitivity is maintained. More work is necessary to clarify whether these are two distinct phenotypes or one phenotype interpreted in two ways.

The data surrounding reductions in catecholamine-induced lipolysis rate elevations are less ambiguous [79]. The balance of surface expression between pro-lipolytic beta-adrenergic receptors and anti-lipolytic alpha-adrenergic receptors in adipocytes may be responsible for this loss of function. Obese adipose tissue from Zucker rats was found to have an increased alpha-adrenergic receptor density [82]. Importantly, weight loss in human subjects is associated with an increased sensitivity to catecholamine-induced lipolysis [83], despite the number of beta-adrenergic receptors being unchanged during weight loss [84]. Disturbances to catecholamine-induced lipolysis have been observed downstream of adrenergic receptor dynamics as well. A decrease in the activity of adenylyl cyclase, which generates cAMP upstream of PKA activation, was detected in obese adipose tissue relative to lean adipose tissue [83]. It is likely that varying degrees of these events exist concurrently and contribute to adipocyte rigidity.

There seems to be a limit to tolerable adipocyte hypertrophy. Upon reaching this threshold, the adipocytes’ ability to store lipids and sensitivity to both lipolytic agonists and antagonists is decreased on the adipose depot level. Due to the fact that the adipose tissue contains a heterogeneous adipocyte population, there is likely variability in the lipid mobilization capacity among individual adipocytes, but the average storage and sensitivity to regulatory stimuli across the organ is lower. More work is required to determine the various lipid mobilization-related phenotypes of adipocytes, such that more effective therapeutic strategies may be developed.

### 3.2. Pathogenic Signaling Patterns

The hormone secretion profile of hypertrophic adipocytes is altered compared with lean adipocytes. Leptin is a pro-lipolytic hormone secreted by adipocytes that acts locally [85,86,87] and on the hypothalamus to reduce food consumption and increase energy expenditure [88]. Leptin-deficient individuals suffer from obesity [89], and the administration of recombinant leptin has been shown to effectively reduce fat tissue mass [90,91]. That said, clinical trials exploring whether the apparent antagonistic relationship between leptin and fat storage may be utilized as treatment for obesity by recombinant leptin administration yielded disappointing results [92]. Compared to lean adipocytes, hypertrophic adipocytes overproduce leptin, directly impairing insulin’s anti-lipolytic effects on PKA-mediated lipolysis [93]. Further, hyperleptinemia and leptin resistance are associated with obesity [92]. A recent study has demonstrated that a partial reduction in leptin levels in obese mice by genetic modulation or monoclonal antibody neutralization ameliorates weight gain and insulin sensitivity [94], suggesting precise leptin lowering strategies may offer benefits to obese individuals.

Adiponectin is another adipose-derived hormone that has been intensely studied since its discovery [95]. Insulin resistance and obesity are inversely correlated with adiponectin in humans. Experiments with rodent models have shown depressed adiponectin production in hypertrophic, rigid adipocytes [88,95]. Mouse models of reduced adiponectin expression are associated with insulin resistance and poor metabolic health [96] whereas adiponectin-overexpressing mice exhibit preserved metabolic health, despite being more obese than their wild-type counterparts [97]. The benefits of adiponectin include increased vascularization [97] and ceramidase activity [98,99]. Adiponectin’s numerous beneficial metabolic effects have recently been reviewed [100] and its insulin-sensitizing mechanisms are still under investigation. A recent report demonstrated that adiponectin treatment increased the LPL activity and TAG uptake in the adipocytes of obese mice. These results correlated with improved metabolic outcomes in insulin-sensitive tissues and on the whole-body level [101]. These studies demonstrate the importance of preserving the adipocyte’s lipid-buffering capacity and ability of the adipose depots to expand [58,102].

In addition to hormonal secretions, adipocytes produce cytokines, enabling autocrine, paracrine, and endocrine communication. Hypertrophic rigid adipocytes are a significant source of potent, pro-inflammatory cytokines, such as tumor necrosis factor alpha (TNFα), interleukin-6, interleukin-1β, and monocyte chemoattractant protein-1 (MCP-1) [103,104]. The deleterious consequences of inflammation in adipose tissue in the cardiometabolic pathology have recently been reviewed [105]. TNFα acts in autocrine and paracrine fashion to inhibit IRS-1 [106], decreasing the insulin efficacy, and to activate MAPKs and JNK, which then downregulate perilipin mRNA and protein [107,108]. Lower levels of perilipin are found in adipose tissue from obese individuals [75]. Importantly, MAPK and ERK1/2 have been shown to phosphorylate and activate HSL in a PKA-independent mechanism [109]. While inflammation does play a role in the pathogenesis of cardiometabolic disorders, clinical trials employing TNFα antagonism as treatment for metabolic syndrome have mostly been unsuccessful in improving insulin sensitivity [110,111], despite evidence that targeting the IKKb/NFkB pathway can lower hyperglycemia [112,113,114]. This suggests that anti-inflammatory efforts alone are not sufficient to restore lost adipocyte phenotype flexibility.

It has long been known that the adipose tissue is not a passive energy storage organ. White adipocyte hormonal and cytokine secretions have demonstrated regulatory abilities related to numerous physiological domains, including satiety, energy expenditure, vascularity, and inflammation. Rigid, hypertrophic adipose tissue has a pathological secretion profile that perpetuates cardiometabolic pathologies.

## 4. How Phenotype Flexibility Is Lost

**Figure 3:**
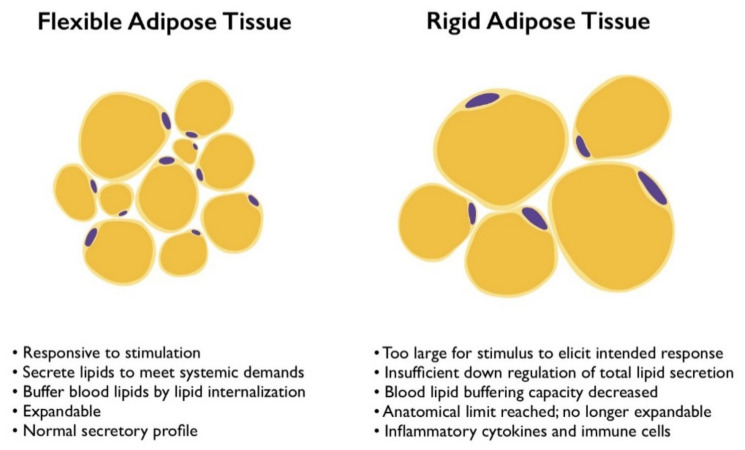
Characteristics of flexible and rigid adipose tissue phenotypes. Adipocyte size is a determinant of lipid-buffering function and responsiveness to stimulation.

Adipose tissue from obese organisms is characterized by metabolic dysfunction, inflammation, fibrosis, and hypoxia (Figure 3) [115]. The sequence of these pathological developments has been the subject of debate for decades, and recent studies have refined our understanding of how adipocytes lose their phenotypic flexibility. When dysfunctional adipocytes render the adipose tissue incapable of buffering lipids, the systemic pathology quickly follows and exacerbates cardiovascular morbidity.

Clinical and animal studies on inflammatory cascade intervention have demonstrated that inflammation is a contributor to cardiometabolic pathologies [105]. Inflammatory signaling [106] and immune cell accumulation [116,117,118,119,120] in adipose tissue cause metabolic dysfunction; however, the initial source of the inflammatory action has remained elusive. Whether the pathogenesis of cardiometabolic disease is initiated by metabolic dysfunction or inflammatory action has long been debated. Substantial evidence now exists indicating that not only does adipocyte metabolic dysfunction occur in the absence of the chronic low-grade inflammation associated with obesity, but the metabolic dysfunction also stimulates immune cell recruitment. In mammals, the target of rapamycin complex 2 (mTORC2) is stimulated by insulin to promote glucose clearance in insulin-sensitive tissues [121,122,123]. The rapamycin-insensitive companion of mTOR (RICTOR) is a component of mTORC2, and the genetic ablation of RICTOR in mice renders them mTORC2-deficient. Mice with adipocyte-specific knockout of RICTOR and mTORC2 deficiency challenged by a high fat diet were insulin-resistant prior to immune cell buildup in the adipose tissue [124]. A diminished insulin sensitivity was associated with an increased expression of the potent leukocyte chemoattractant, MCP1, and recruitment of M1 macrophages. While inflammatory action has been shown to affect insulin sensitivity in various ways, this study unveils a model of pathogenesis in which inflammation is a contributor, but not the initiator, of pathogenesis.

Efforts have been made to determine a causal relationship between adipocyte hypertrophy and metabolic dysfunction. Cell culture experiments have demonstrated that 3T3-L1 adipocytes become hypertrophic when stimulated with saturated or monounsaturated fatty acids, but only saturated fatty acid treatment increased pro-inflammatory cytokine mRNA levels and secretion [125]. Despite the absence of an inflammatory reaction, hypertrophic adipocytes stimulated with monounsaturated fatty acid exhibited insulin resistance comparable to saturated fatty-acid-stimulated hypertrophic adipocytes. To corroborate this data, mice deficient in Toll-like receptor 4 were fed a high fat diet that caused adipocyte expansion and insulin resistance in the absence of inflammation [125]. Analyses of human adipose tissue biopsies have shown that adipocyte hypertrophy in the visceral adipose depot, specifically, is a close correlate to whole-body insulin sensitivity, regardless of the local immune cell activity [126]. Further, the “metabolically healthy obese” phenotype (reviewed in [127]), which has been regarded as benign, has recently been shown to be associated with a higher long-term risk of cardiovascular disease and metabolic syndrome than lean counterparts [128,129].

While the insights gained from these studies are valuable, it remains unclear whether hypertrophic adipocytes alone are a significant enough perturbation to metabolic homeostasis to cause pathology. Mice overexpressing adiponectin are protected against the inflammatory and metabolic consequences of severe obesity [97]. The adipose tissue capillary density of diet-induced obese (DIO) adiponectin transgenic mice was significantly higher than in wild-type controls, suggesting that tissue hypoxia may play a role. In addition, the treatment of type 2 diabetes with thiazolidinediones, a class of drug targeting peroxisome proliferator-activated receptor-γ (PPARγ), increases insulin sensitivity but is paradoxically associated with adipose tissue expansion and weight gain [130,131]. This phenomenon is purportedly owed to induced adipogenesis [132]. These observations suggest that an inability of adipocytes to expand, a natural consequence of adipocyte hypertrophy, may be the root cause of adipocyte dysfunction. Fat-specific protein 27 (Fsp27) is a lipid-droplet-associated protein that regulates unilocular lipid droplet formation through interactions with perilipin1 [133]. Mice lacking adipocyte-specific Fsp27 challenged with a high fat diet had a decreased lipid clearance from the blood and reduced adipose tissue expansion, resulting in severe hepatic lipotoxicity and metabolic dysfunction, all in the absence of adipose tissue inflammatory remodeling [134]. These data emphasize the importance of preserving the adipocyte’s lipid-buffering capacity [58].

The aforementioned studies do not address the question of lean adipocyte dysregulation, which likely occurs before the manifestation of obesity. To define the initial steps of adipose tissue dysfunction, the postprandial phase must be observed in lean, healthy subjects. Human studies have revealed that the acute consumption of saturated fatty acid [135] and monounsaturated fatty acid [136] results in systemic, hepatic, and adipose tissue insulin resistance during the postprandial phase in healthy volunteers. A similar rapid induction of insulin resistance has been observed in skeletal muscle, although saturated fatty acids induced higher levels of ceramides and activated different protein kinase C isoforms compared to monounsaturated fatty acids, a possible explanation for discrepancies in long-term outcomes [137]. It is hypothesized that repetitive nutrient overload is the impetus for cardiometabolic pathogenesis. Lean mice fed single high-fat meals undergo a transient inflammatory response in the visceral adipose tissue that is extended with continued high-fat feeding [120]. Perhaps the most convincing evidence that the chronic overfed state is responsible for pathogenesis comes from studies employing intermittent fasting. Disrupting ad libitum access to a high-fat diet prevents mice from developing obesity, hyperinsulinemia, hepatic steatosis, and inflammation, despite eating equivalent calories [138,139]. Preliminary reports on the effectiveness of intermittent fasting as a treatment for patients with metabolic syndrome are promising [140], and clinical work to corroborate these preliminary data is underway [141].

Insufficient oxygen delivery to adipocytes is a likely contributor to metabolic dysregulation. Exposing mice to chronic intermittent [142] and acute hypoxia [143] increases adipocyte lipolysis by increased sympathetic stimulation and decreases lipid uptake by limiting LPL activity. Unfortunately, efforts to reproduce these phenomena in lean humans have been unsuccessful [144]. Nonetheless, hypoxia due to insufficient angiogenesis and oxygen supply to match the adipocyte demand has long been regarded a driver of pathological adipose tissue remodeling. Indeed, increasing angiogenesis in DIO mice by overexpressing adiponectin has been shown to improve metabolic outcomes [97]. Tissue oxygenation is dependent on both oxygen supplied to the tissue by vasculature and the tissue metabolic demand for oxygen. Insufficient neovascularization to match adipose tissue expansion and to adequately deliver oxygen results in tissue hypoxia, increased inflammatory action, and reduced insulin sensitivity [145,146,147]. The question of whether variations in adipocyte oxygen demand, independent of vascular density, drive adipocyte dysfunction has more recently been addressed [148,149]. Accumulated intracellular saturated fatty acids have been proposed to stimulate uncoupled mitochondrial respiration, increasing the oxygen consumption and HIF1-alpha expression, in an adenine nucleotide transporter-2 (ANT2)-dependent fashion [148]. ANT2 is a mitochondrial protein involved in the exchange of ATP and ADP across the mitochondrial inner membrane. The genetic knockdown of adipose tissue ANT2 in mice demonstrated that obese adipocytes are under a significantly higher oxygen demand relative to lean adipose samples. Adipocyte hypoxia, therefore, precedes adipose tissue hypoxia, and targeting adipocyte ANT2 expression ameliorated adiponectin levels, insulin sensitivity, and inflammatory markers [149]. These data suggest that the unchecked accumulation of saturated fatty acids in the adipocyte is, in itself, pathological, and that this process may be a candidate for early intervention in pathogenesis.

While inflammation has been shown to contribute to metabolic dysfunction, recent work has demonstrated that metabolic dysregulation may precede and even cause energy-imbalance-related inflammatory action [124]. Further, experiments with 3T3L1 adipocytes have shown that adipocyte hypertrophy and metabolic dysfunction occur in the absence of inflammation when treated with monounsaturated fatty acids [125]. The relationship between hypertrophic adipocytes and metabolic function does not explain observable disturbances in metabolism and inflammation that occur in lean individuals [135,136] and rodents [120]. In mice, the ultimate culprit appears to be frequent, repetitive high-fat feeding [138]. Time-restricted feeding ameliorates high-fat feeding outcomes in mice [138] and humans [140]. The mechanistic explanation for why intermittent fasting has so far been effective may be related to an improved overall energy balance and decreased adipocyte hypoxia due to a reduced accumulation of saturated fatty acids [148,149].

## 5. Conclusions

The adipose tissue’s unique ability to secrete and internalize lipids is dependent upon its phenotypic flexibility. A chronic energy imbalance associated with adipocyte hypertrophy narrows the adipose tissue’s range as a source of or reservoir for lipids, resulting in hyperlipidemia, lipotoxicity, and obesity. To this end, dysfunctional lipolysis and lipid clearance are often discussed as physiological points of intervention. Anti-lipolytic strategies have been suggested, with the goal of reducing hyperlipidemia; however, a way to make the hypertrophic adipose tissue, which already has diminished responses to lipolytic stimuli, even less responsive, would be to increase the adipocyte rigidity. On the other hand, increasing lipolytic rates without also increasing the energy expenditure may increase the lipotoxicity. Efforts to improve the adipose tissue lipid clearance and adipocyte expandability may improve lipid levels, but will also increase the total adiposity, as is observed with thiazolidinedione treatment. The efficacy of intervening in lipid mobilization processes will ultimately depend upon restoring the energy balance.

Novel strategies to aid restoring the energy balance in patients with a high incidence of cardiometabolic risk factors are still needed. Obesity is associated with hyperleptinemia and leptin resistance. Paradoxically, reducing leptin levels in mouse models of obesity improved metabolism, suggesting a new hormonal technique for combatting disease. Adiponectin administration may also be valuable in improving metabolic outcomes; however, increasing the adiposity of overweight individuals may prove counterproductive. Anti-inflammatory approaches alone appear insufficient in improving cardiometabolic outcomes; however, whether their use in conjunction with other techniques is effective remains unclear. Recent studies have revealed that adipocyte hypoxia due to saturated fatty acid accumulation may be an early driver of metabolic dysfunction. To that end, ANT2 inhibition may aid efforts to improve metabolic homeostasis in hypertrophic adipose tissue. Rodent and human studies employing time-restricted feeding have shown promise. This technique addresses the core problem of adipocyte hypertrophy, energy imbalance, and likely ameliorates several molecular mechanisms simultaneously.

Pathological lipid dysregulation is a risk factor for the development of metabolic syndrome, type 2 diabetes, nonalcoholic fatty liver disease, and atherogenesis. The cardiometabolic disease epidemic is likely to become more severe before improving, as the rate of new cases has not slowed and society is moving further into sedentary lifestyles. A better understanding of how phenotypically rigid adipose tissue manifests will aid scientists and clinicians in identifying therapeutic strategies for the treatment of obesity-related pathologies and will provide guidance on the prevention of new cases. Rodent models have made clear that adipocyte metabolic dysfunction, tissue hypoxia, and inflammation work in concert to render adipose tissue incapable of buffering blood lipids. The loss of adipose-dependent lipid homeostasis regulation causes systemic metabolic disease. Re-establishing the adipose tissue lipid uptake in obese humans has reliably improved metabolic outcomes (thiazolidinediones, recombinant adiponectin), but new techniques and strategies are required.
